# Molecular association and morphological characterisation of *Himalopsyche* larval types (Trichoptera, Rhyacophilidae)

**DOI:** 10.3897/zookeys.773.24319

**Published:** 2018-07-09

**Authors:** Anna E. Hjalmarsson, Wolfram Graf, Sonja C. Jähnig, Simon Vitecek, Steffen U. Pauls

**Affiliations:** 1 Goethe-University Frankfurt, 60323 Frankfurt am Main, Germany. Senckenberg Research Institute and Natural History Museum, Senckenberganlage 25, 63025 Frankfurt am Main, Germany; 2 Institute of Hydrobiology and Aquatic Ecology Management, University of Natural Resources and Life Sciences, Gregor-Mendel-Straße 33/DG, 1180 Vienna, Austria; 3 Leibniz-Institute of Freshwater Ecology and Inland Fisheries (IGB), Department of Ecosystem Research, Justus-von-Liebig-Str. 7, 12489 Berlin, Germany; 4 Senckenberg Research Institute and Natural History Museum, Senckenberganlage 25, 63025 Frankfurt am Main, Germany; 5 Anna E. Hjalmarsson

**Keywords:** Caddisfly, GMYC, Hengduan Mountains, Himalaya, life stage association, PTP

## Abstract

*Himalopsyche* Banks, 1940 (Trichoptera, Rhyacophilidae) is a genus of caddisflies inhabiting mountain and alpine environments in Central and East Asia and the Nearctic. Of 53 known species, only five species have been described previously in the aquatic larval stage. We perform life stage association using three strategies (GMYC, PTP, and reciprocal monophyly) based on fragments of two molecular markers: the nuclear CAD, and the mitochondrial COI gene. A total of 525 individuals from across the range of *Himalopsyche* (Himalayas, Hengduan Shan, Tian Shan, South East Asia, Japan, and western North America) was analysed and 32 operational taxonomic units (OTUs) in our dataset delimited. Four distinct larval types of *Himalopsyche* are uncovered, and these are defined as the *phryganea* type, *japonica* type, *tibetana* type, and *gigantea* type and a comparative morphological characterisation of the larval types is presented. The larval types differ in a number of traits, most prominently in their gill configuration, as well as in other features such as setal configuration of the pronotum and presence/absence of accessory hooks of the anal prolegs.

## Introduction

With ~15,000 described and around 50,000 presumed species, caddisflies are one of the larger insect orders and the largest primary aquatic insect order (de Moor and Ivanov 2008). Trichoptera have merolimnic life histories and the larvae have long been recognised as important ecological indicators (Resh and Unzicker 1975). They have several remarkable ecological traits, among others their diverse case-building behaviour or their ability to invade all types of aquatic habitats across the globe (except Antarctica; Holzenthal et al. 2007, Wiggins 1996). While renowned for their intricate cases, larvae of some caddis families roam freely, and only build pupal retreats. Among those, the family Rhyacophilidae Stephens, 1836 is particularly noteworthy for their diversity and ecological differentiation. This family entails the genera *Himalopsyche* Banks, 1940, *Fansipangana* Mey, 1996, *Philocrena* Lepneva, 1956, *Phoupanpsyche* Malicky, 2008, and *Rhyacophila* Pictet, 1834.

Species of the genus *Himalopsyche* are a particularly interesting group of caddisflies. They primarily inhabit mountain and alpine environments in Central and East Asia, although the genus also radiated into the Nearctic where it is represented by a single species, *H. phryganea* (Ross, 1941). *Himalopsyche* larvae mostly inhabit highly turbulent, fast-flowing streams, where they live as ferocious predators.

Including recent species descriptions, the genus *Himalopsyche* currently comprises 53 known species, adding to the status of the last major treatment of *Himalopsyche* by Schmid and Botosaneanu (1966). While our knowledge of the adult taxonomy of *Himalopsyche* is comparatively good, we know very little about the larval taxonomy and ecology of individual species. To date, five species have been described in the larval stage: *H. japonica* (Morton, 1900), *H. phryganea*, *H. gigantea* (Martynov, 1914), *H. tibetana* (Martynov, 1930), and *H. acharai* Malicky & Chantaramongkol, 1989 (Flint 1961, Graf and Sharma 1998, Lepneva 1945, 1970, Saito 1965, Tanida 1985, Thamsenanupap et al. 2005), as well as a larva corresponding to a hitherto unknown species, referred to as *H*. ‘larva hoplura’ (Lepneva 1945, 1970). Three distinct types of *Himalopsyche* larvae have been differentiated, most prominently based on their gill configuration, but also on differences in setal configuration of the pronotum and anal sclerites. In their comparative study, Graf and Sharma (1998) defined two of these larval types, Type A and Type B, which differ distinctly from the previously known larvae of *H. phryganea* and *H. japonica*. Type B could be assigned to *H. tibetana*. The species identity of larvae assigned to Type A could not be clarified before now, but this type shows similarities to larvae of *H. gigantea*. The larva of *H. acharai* was described by Thamsenanupap et al. (2005) and was compared to *H. japonica* and *H. phryganea*, but not with Type A and B *sensu* Graf and Sharma (1998). Thus, all *Himalopsyche* species known in the larval stage have never been compared and characterised simultaneously before.

Caddisflies are good biological indicators (e.g., Rainbow et al. 2012) and are essential elements in many standardised assessment systems, especially in North America, Europe, and Australia (e.g., Schmidt-Kloiber et al. 2008). However, the practical use of Trichoptera larvae as biological indicators is limited whenever taxonomic knowledge is poor. Higher-level taxonomic resolution (e.g., genus or family level) can often mask the variability of environment/species interactions that act at the species level and make these taxa valuable indicator species (Resh and Unzicker 1975, Verdonschot 2006, Ruiter et al. 2013).

Molecular data have proven successful in facilitating the association of larvae with adults in Trichoptera (e.g., Graf et al. 2005, Ruiter et al. 2013, Zhou et al. 2007). Since the earliest studies of this kind (Graf et al. 2005, Zhou et al. 2007) the mitochondrial cytochrome c oxidase (COI) and nuclear rDNA 28S genes have been the markers of choice for life stage associations. Zhou et al. (2007) suggested a protocol for life stage association based on COI (439 bp) and 28S (~430 bp) and emphasised that it is important to use more than a single genetic marker for life stage association. There are several reasons for this. First, data from different genes provide the possibility to cross-validate the results from each gene, and to identify potential contamination issues. Second, gene trees are likely to differ from the species phylogeny among close relatives because of incomplete lineage sorting (Funk and Omland 2003). Using more than one gene therefore increases the opportunities to cross-validate results from one gene with the other gene(s).

In this study, we associate larvae with adults based on molecular data from a nuclear and a mitochondrial gene, CAD and COI, respectively, and employ three different methods for life stage association to generate a consensus result. We then present the first comparative morphological characterisation of all known larval types of *Himalopsyche*.

## Materials and methods

### Sampling

**Figure 1.**
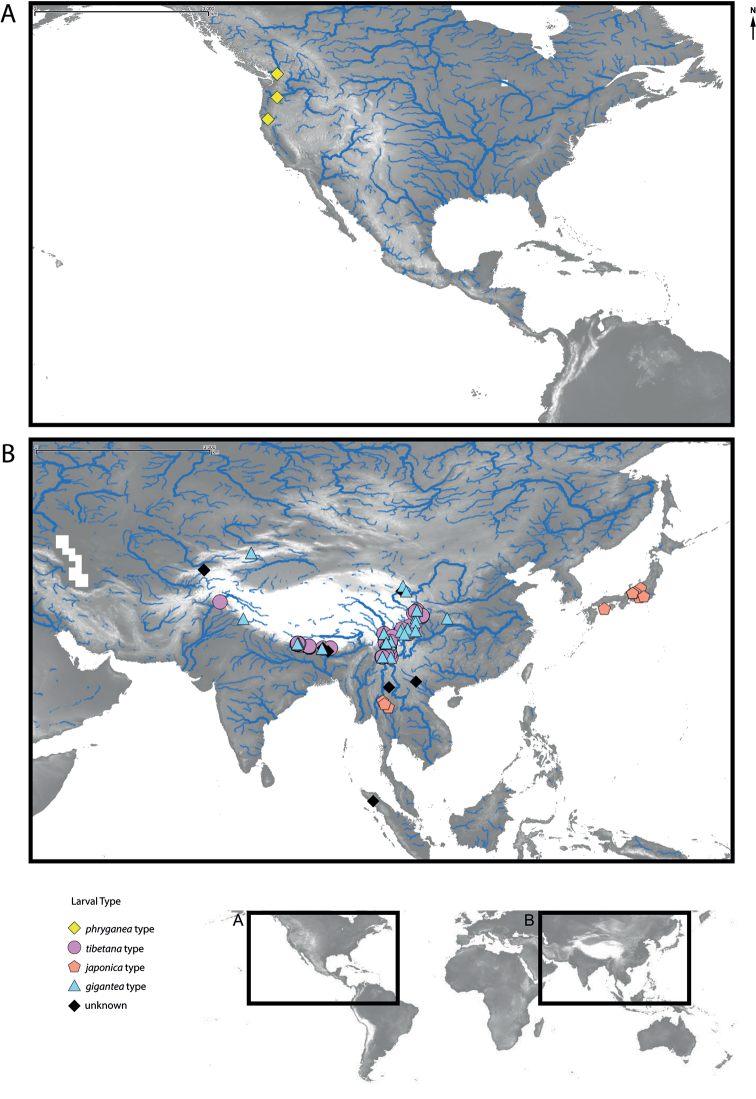
Map showing sampling localities in **A** North America, and **B** East Asia. Colours/symbols indicate larval types of the OTUs. Elevation data from Jarvis et al. (2008), stream data from Andreadis et al. (2013).

Larval specimens used in this study were mostly collected in Nepal and China in 2011–2013; adult material was largely obtained from research collections (Suppl. material 1). In Nepal, samples were collected in 2012 and 2013 from the Langtang, Indrawati, and Arun catchments (Tachamo Shah et al. 2015) and in China from the Lancang and Jinsha catchments in 2011 and 2013 (Figure 1). Larvae were collected from streams using hand nets, kick nets and hand-picking. Adults were collected using light traps, and occasionally with hand nets and a Malaise trap. Light traps were either set up at dusk, using a white sheet and a UV lamp (active light trapping), or left over night with the lamp placed over a small pan filled with soap water (passive light trapping; Blahnik and Holzenthal 2004, Malicky 2004). Lamps used included 12V 15W Bioform blacklights, F8W/T5/BL350 Sylvania blacklights, and 22W circline BioQuip blacklights. All specimens were stored directly in 95% ethanol in the field, and refrigerated in the lab. Museum specimens of adults were borrowed for DNA sequencing from the private collection of Hans Malicky (Lunz am See, Austria), and from the Museum für Naturkunde (Berlin, Germany).

### Molecular data

For this study, we used partial sequence data of the single copy nuclear marker CAD and the mitochondrial COI. CAD has proved useful for insect phylogenetics (Bukontaite et al. 2014, Ekrem et al. 2010, Johanson and Malm 2010, Klopfstein et al. 2013, Moulton and Wiegmann 2004, Sharanowski et al. 2011, Wild and Maddison 2008) and in species delimitation (Foster et al. 2013, Song and Ahn 2014, Vitecek et al. 2017). For COI, we targeted the 658 bp “standard barcode region” fragment of COI that has been used extensively for species identification, but also for life stage association, population genetics, and phylogeny in insects (e.g., Hebert et al. 2004, Hjalmarsson et al. 2015, Ruiter et al. 2013, Zhou et al. 2007, 2016).

DNA was extracted from legs using one of the following methods: HotShot protocol (Montero-Pau et al. 2008; mainly used for larvae), Qiagen Dneasy Blood & Tissue Kit (Qiagen, mainly used for fresh adult material), or QIAamp DNA Micro Kit (Qiagen, mainly used for museum material). We used a combination of previously published and newly developed primers, specific to *Himalopsyche*. The primers for CAD were: 743nF-ino & 1028r-ino (850 bp; Johanson and Malm 2010) and C1Fb & C7Ra (758 bp; this work, Table 1). The COI primers were: HCO1490 &LCO2198 (658 bp; Folmer et al. 1994), and B1Fa & B3Ra (367 bp; this work, Table 1). Polymerase Chain Reaction (PCR) was performed using PeqGOLD Hot Start Taq Polymerase kits (PeqLab VWR) in standard reactions, for some protocols with the addition of BSA (Table 2). Sanger sequencing of PCR products was performed on a 3730XL DNA Analyzer (Applied Biosystems) at the Senckenberg Biodiversity Climate Research Centre Laboratory Centre. Sequences were assembled and edited in Geneious 7.0.6 (Kearse et al. 2012). Ambiguities were coded using IUPAC codes. Multiple sequence alignments of CAD and COI were made using the ClustalW algorithm (Thompson et al. 1994) as implemented in Geneious, and were checked for stop codons.

**Table 1.** Primers used for PCR and sequencing. Fragment lengths refer to the primer pairs 743nF-ino & 1028r-ino, C1Fb & C7Ra, HCO1490 & LCO2198, and B1Fa & B3Ra.

Gene	Primer	Sequence	Tm (°C)	Fragment length (bp)	Reference
CAD	743nF-ino	5’-GGIGTIACIACIGCITGYTTYGARCC-3’	52.4	850	Johanson and Malm 2010
CAD	1028r-ino	5’–TTRTTIGGIARYTGICCICCCAT–3’	42.1	850	Johanson and Malm 2010
CAD_internal_	C1Fb	5’–TGYGTTGTRAAGATTCCGAG-3’	51.8	736	this work
CAD_internal_	C7Ra	5’–TGTCCATTACAACCTCGAATG-3’	62.3	736	this work
COI	HCO1490	5’-GGTCAACAAATCATAAAGATATTGG-3’	53.2	658	Folmer et al. 1994
COI	LCO2198	5’-TAAACTTCAGGGTGACCAAAAAATCA-3’	51.0	658	Folmer et al. 1994
COI_internal_	B1Fa	5’-ATTGCDACWGATCAWACAAA-3’	54.9	367	this work
COI_internal_	B3Ra	5’-AAYGTARTWGTWACWGCTCA-3’	47.2	367	this work

**Table 2.** Protocols for 10 μL PCR reactions, using VWR peqGOLD Hot Taq DNA Polymerase kits. BSA = Bovine serum albumin. All numbers are given in μL.

Gene	Primer pair	Buffer S	Buffer Y	dNTPS 2mM	BSA 20 mg/ml	Forward primer 10μM	Reverse primer 10μM	Taq	DNA	H_2_O	Cycler
CAD	1028r-ino, 743nF-ino	1	0	1	0	0.25	0.25	0.1	1	6.4	5’ 95°C, 35x (45’’ 95°C, 45” 55°C, 60” 72°C) 5’ 72°C
CAD	C1Fb, C7Ra	1	0	1	0	0.25	0.25	0.1	1	6.4	5’ 95°C, 35x (30’’ 95°C, 30” 50°C, 45” 72°C) 5’ 72°C
COI	HCO1490, LCO2198	1	0	1	0	0.25	0.25	0.1	1	6.4	5’ 95°C, 5 x (30’’ 95°C, 1’ 44°C, 1’ 72°C), 15x (30’’ 95°C, 30’’ 48°C, 1’ 72°C), 20 x (30’’ 95°C, 30’’ 50°C, 1’ + (10’’ * n) 72°C), 5’ 72°C
COI	B1Fa, B3Ra	1	0	1	0.4	0.25	0.25	0.1	1	6.0	5’ 95°C, 35x (30’’ 95°C, 30” 45°C, 45” 72°C) 5’ 72°C

**Figure 2.**
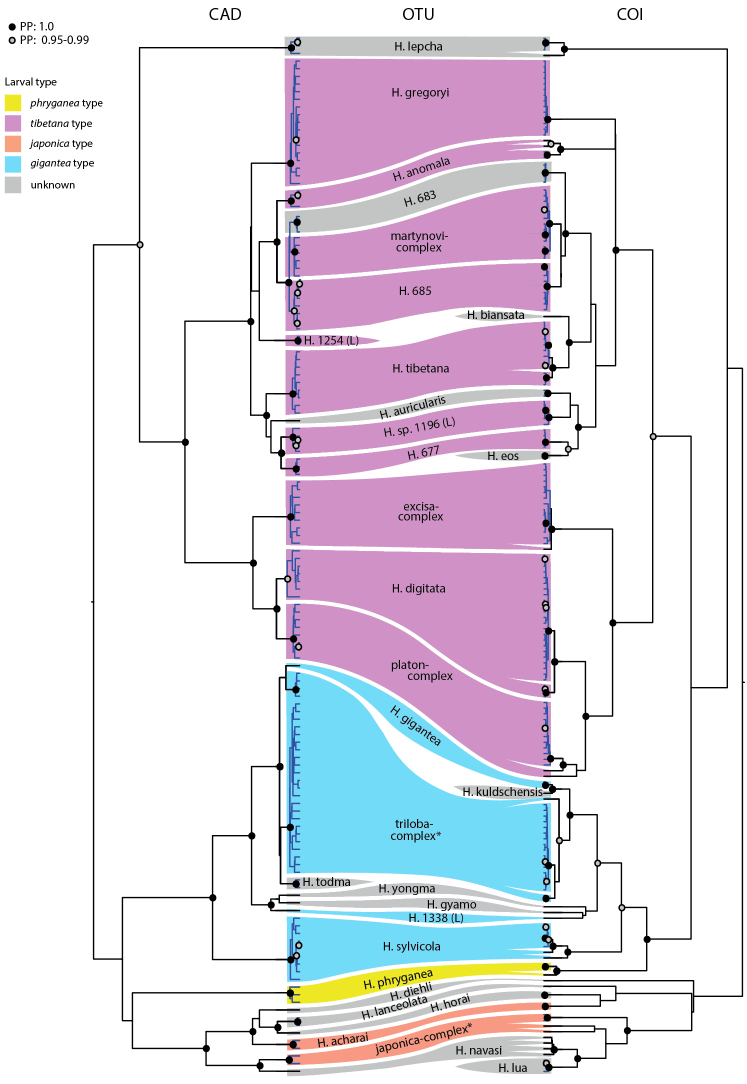
Summary of GMYC results from the CAD and COI, indicating the overlap in results from the two genes. Colours indicate larval types, inferred from this study and the literature.

### Life stage association

Adult males were identified to species based on morphology. The dataset included 38 adult species based on males, including four putative new species (Hjalmarsson submitted, Kuranishi et al. unpublished data). We used and compared the results of three phylogenetic association criteria: Poisson Tree Process (PTP; Zhang et al. 2013), General Mixed Yule Coalescent method (GMYC; Fujisawa and Barraclough 2013, Pons et al. 2006), and reciprocal monophyly. In total, we had five life stage association criteria (PTP and GYMC for each gene, and reciprocal monophyly). Life stage association was considered successful if at least three criteria were fulfilled. The life stage association criteria were defined as follows. PTP: larvae are conspecific with an adult if they form a PTP cluster containing only one and the same adult species. GMYC: larvae are conspecific with adults if they form a GMYC cluster containing only one and the same adult species. Reciprocal monophyly: species are considered reciprocally monophyletic if both genes return a monophylum containing one and the same adult species, and the same larval specimens. Reciprocal monophyly could only be tested for species with data from both genes and with >1 specimen per gene. We considered nodes as supported if they had posterior probability of at least 95%. We refer to groups of unresolved and paraphyletic species as ‘species complexes’.

Gene trees of all available specimens were reconstructed in MrBayes v3.2.6 (Ronquist et al. 2012). Alignments were partitioned per codon position with independent rates among partitions. Nucleotide substitution models were determined in PartitionFinder v2.1.1. (Lanfear et al. 2016; Table 3). Runs were generated for 10 to 50 million generations and were checked for convergence in Tracer v.1.6 (Rambaut et al. 2014). Fully resolved 50% majority rule consensus trees were generated using the ‘sumt’ command, with 25% burn-in. PTP and test of reciprocal monophyly were performed on the gene trees from MrBayes.

**Table 3.** Specifications of alignments used for gene tree reconstruction with BEAST and MrBayes.

Alignment	Number of sequences	Length (bp)	Variable sites	Parsimony informative sites	Missing data	Analysis	Substitution model
CAD	353	736	37,6%	28,0%	1.5%	MrBayes	1: GTR+I2: F813: HKY+G
CAD haplotypes	136	736	31.8%	26.4%	1.6%	BEAST	1: BMod2: BMod3: BMod
COI	451	658	40,0%	37,7%	18.5%	MrBayes	1: SYM+I+G2: F81+I3: GTR+I+G
COI haplotypes	183	658	39.2%	35.0%	15.9%	BEAST	1: BMod2: BMod3: BMod

The GMYC method uses a haplotype-based ultrametric gene tree to determine the transition from inter- to intraspecific branching patterns (Fujisawa and Barraclough 2013; Pons et al. 2006). For this, we reconstructed chronograms in BEAST (see below). GMYC was performed separately on chronograms from each gene in R 3.2.3 (R Core Team 2015), using the *splits* package (Ezard et al. 2014). The single-threshold option of GMYC was used. It sets a single limit between inter- and intraspecific divergence patterns and has been shown to outperform the multiple-threshold option (Fujisawa and Barraclough 2013).

Haplotype-based chronograms require that identical sequences be removed from the alignment, leaving an alignment consisting only of unique sequences. Identical haplotypes were removed from the original alignments using collapsetypes_v4.6 (Chesters 2013), which outputs a reduced fasta-alignment and haplotype assignations of each sequence. Ultrametric gene trees based on haplotype alignments were reconstructed in BEAST2 v. 2.3.1 (Bouckaert et al. 2014). Model selection was done using bModeltest (Bouckaert and Drummond 2015), which estimates the best fitting model of sequence evolution simultaneously with the Bayesian tree search. The transition/transversion split option was chosen, which searches among 31 models of sequence evolution. The ‘empirical’ option was used for base frequencies. Alignments were partitioned per codon position with independent rates among partitions; trees and clocks were linked among partitions. Trees were reconstructed under a relaxed lognormal clock, and with a coalescent constant population tree prior. Priors were set to default settings, with infinity values replaced with hard bounds at 1000, to avoid improper priors. Independent analyses were executed with a run time of 0.5 to 1 billion generations each and runs were checked in Tracer. Maximum clade credibility trees were generated in TreeAnnotator, with 10–50% burn-in. PTP and GMYC analyses were performed on two independent trees, to check stability of the results. *Rhyacophila polonica* McLachlan, 18*79 w*as included as outgroup for MrBayes analyses but was not included in BEAST analyses.

For this paper, we define the use of the terms ‘species’, ‘putative new species’, ‘cluster’ and ‘OTU’ (operational taxonomic unit) as follows: ‘Species’ refers to formally described morphological taxa, following established taxonomy. With ‘putative new species’ we mean morphologically distinct taxa that are still unknown in the literature. The term ‘cluster’ refers to specific results from one of the two analyses, outputting delimited GMYC and PTP ‘clusters’, respectively. For our consensus result from morphology, PTP, and GMYC based on CAD and COI we use the term OTU, which can represent single species or groups of species referred to as species complexes.

### Comparative morphological studies

Comparative morphological analysis of larvae followed a standard procedure. We screened all larvae of each OTU for consistent morphological characters. Instar differentiation and thus assignment of most larvae to different instars is not possible with the currently available material. Therefore, general features commonly represented by all OTUs within one phylogenetic clade were considered as synapomorphies. Some characters were found present across all size classes of single OTUs and phylogenetic clades, e.g., distolateral accessory hooks on lateral plates of anal prolegs were consistently present in even the smallest instars.

## Results

### Datasets

The dataset comprised 525 *Himalopsyche* individuals (205 adults, 313 larvae and 8 pupae), and *R. polonica* as outgroup for MrBayes. We generated 352 *Himalopsyche* sequences of CAD (736 bp) and 450 *Himalopsyche* sequences of COI (658 bp). After haplotype reduction of the alignments, the CAD alignment had 136 unique haplotypes and the COI had 183 unique haplotypes. The total CAD alignment had 37.6% variable sites; the total COI haplotype alignment had slightly more with 40% (Table 3). For morphological treatment, we also included larvae collected in Japan and Thailand identified as *H. japonica* and *H. acharai*, respectively, although no molecular data for these specimens were available.

### Life stage association

PTP delimited 29 clusters with CAD, and 62 with COI for the ingroup. GMYC delimited 27 clusters with CAD and 46–48 clusters with COI. The results from separate runs were stable except for GMYC with COI. This instability did not affect larval association and we hereafter only refer to COI run 1 which delimited 48 GMYC clusters (Table 4, Suppl. material 2, 3). We defined OTUs based on adult male morphospecies to the extent that it was possible. Clades containing several paraphyletic species were grouped into ‘species complexes’. In all cases but two, COI clusters were nested within CAD clusters, yielding an overall compatible result. The exceptions were the OTUs *japonica*-complex (*H. japonica* and a putative new Japanese species [*H*. sp. n. 1529]), and *triloba*-complex (*H. triloba* (Hwang, 1958), *H. efiel* Malicky, 2012, *H. hageni* Banks, 1940, *H. malenanda* Schmid, 1963, *H. maculipennis* (Ulmer, 1905a), and *H. yatrawalla* Schmid & Botosaneanu, 1966), which both were paraphyletic in COI. The remaining species complexes were: *martynovi*-complex (*H. martynovi* Banks, 1940, *H. epikur* Malicky, 2011), and *excisa*-complex (*H. excisa* Ulmer, 1905b, *H. placida* Banks, 1947 and *H. maitreya* Schmid, 1963). Three OTUs were identified for which only larval material was available: *H*. sp. 1196 (L), *H*. sp. 1338 (L), and *H*. sp. 1254 (L). A *H. platon* Malicky, 2011 male formed a clade together with samples of larvae and females in COI but we lacked CAD data from the adult male so we cannot at this stage conclude whether this monophylum constitutes one or several species, and refer to this monophylum as *platon*-complex.

**Table 4.** Number of clusters delimited by PTP and GMYC. Number of PTP clusters refer to the ingroup only.

Gene	Method	Run	Chain length	Burn-in	ESS	Clusters
CAD	MrBayes & PTP	1	1* 10^7^	25%	All >200	29
CAD	MrBayes & PTP	2	1* 10^7^	25%	All >200	29
CAD	BEAST & GMYC	1	1* 10^9^	50%	All >200	27
CAD	BEAST & GMYC	2	5 * 10^8^	10%	Most >200	27
COI	MrBayes & PTP	1	5* 10^7^	25%	All >200	62
COI	MrBayes & PTP	2	1* 10^7^	25%	Most >200	62
COI	BEAST & GMYC	1	1* 10^9^	10%	All >200	48
COI	BEAST & GMYC	2	1 * 10^9^	10%	All >200	46

We could unambiguously associate 239 larvae and eight pupae to the following nine species: *H. acharai*, *H. anomala* Banks, 1940, *H. digitata* (Martynov, 1935), *H. gregoryi* (Ulmer, 1932), *H. phryganea*, *H. sylvicola*, *H. tibetana*, *H. 677*, and *H. 685* (Table 5, Suppl. material 1). We could additionally associate 65 larvae to OTU-level for seven OTUS: *excisa*-complex, *martynovi*-complex, *platon*-complex, *H*. sp. 1196 (L), *H*. sp. 1254 (L), *H*. sp. 1338 (L), and *triloba*-complex.

**Table 5.** Overview of life stage association results for each OTU. Asterisks denote. The OTUs *martynovi*-complex, *H. 683*, and *H. 685*, formed a single GMYC cluster in CAD, here referred to as ‘*martynovi*-clade’. Key: ^†^ indicates larvae without DNA data, * indicates conflicts in COI and CAD gene tree topologies.

OTU	Samples	Species	Larvae	Larval type	CAD units (PTP/GMYC)	COI units (PTP/GMYC)	Association with CAD (PTP/GMYC)	Association with COI (PTP/GMYC)	Reciprocal monophyly (both genes)	Number of criteria fulfilled
*H. acharai*	4	*H. acharai*	yes	*japonica* type	1/1	1/1	yes/yes	yes/yes	yes	5
*H. anomala*	6	*H. anomala*	yes	*tibetana* type	1/1	4/3	yes/yes	no/yes	yes	4
*H. auricularis*	2	*H. auricularis*	no	unknown	1/1	2/1	–	–	not applicable	–
*H. biansata*	2	*H. biansata*	no	unknown	no data	1/1	–	–	not applicable	–
*H. diehli*	1	*H. diehli*	no	unknown	1/1	1/1	–	–	not applicable	–
*H. digitata*	34	*H. digitata*	yes	*tibetana* type	2/1	2/2	yes/yes	no/no	yes	3
*H. eos*	2	*H. eos*	no	unknown	no data	1/1	–	–	not applicable	–
*excisa*-complex	31	*H. excisa, H. placida, H. maitreya*	yes	*tibetana* type	1/1	5/2	no/no	no/no	no	0
*H. gigantea*	2	*H. gigantea*	no	*gigantea* type	no data	1/1	–	–	not applicable	–
*H. gregoryi*	77	*H. gregoryi*	yes	*tibetana* type	1/1	1/1	yes/yes	yes/yes	yes	5
*H. gyamo*	1	*H. gyamo*	no	unknown	1/1	1/1	–	–	not applicable	–
*H. horai*	5	*H. horai*	no	unknown	1/1	1/1	–	–	yes	–
*japonica*-complex*	8	*H. japonica, H*. sp. n. 1529	yes^†^	*japonica* type	2/1	4/3	–	–	no	–
*H. kuldschensis*	2	*H. kuldschensis*	no	unknown	1/1	1/1	–	–	yes	–
*H. lanceolata*	4	*H. lanceolata*	no	unknown	1/1	1/1	–	–	yes	–
*H. lepcha*	5	*H. lepcha*	no	unknown	1/1	2/2	–	–	yes	–
*H. lua*	5	*H. lua*	no	unknown	no data	2/1	–	–	not applicable	–
*martynovi*-complex	30	*H. martynovi, H. epikur*	yes	*tibetana* type	*martynovi*–clade	1/1	no/no	no/no	no	0
*H. navasi*	4	*H. navasi*	no	unknown	1/1	4/4	–	–	not applicable	–
*H. phryganea*	4	*H. phryganea*	yes	*phryganea* type	1/1	2/2	yes/yes	yes/yes	yes	5
*platon*-complex	27	*H. platon*, *H.*. sp (F), *H.*. sp (L)	yes	*tibetana* type	1/1	4/3	no/no	no/no	yes	1
*H*. sp. 1196 (L)	17	NA	yes	*tibetana* type	1/1	2/1	–	–	yes	–
*H.* sp. 1254 (L)	4	NA	yes	*tibetana* type	1/1	no data	–	–	not applicable	–
*H*. sp. 1338 (L)	1	NA	yes	*gigantea* type	1/1	1/1	–	–	not applicable	–
*H. sylvicola*	33	*H. sylvicola*	yes	*gigantea* type	1/1	6/3	yes/yes	no/yes	yes	5
*H. tibetana*	58	*H. tibetana*	yes	*tibetana* type	1/1	2/2	yes/yes	yes/yes	yes	5
*H. todma*	4	*H. todma*	no	unknown	1/1	no data	–	–	not applicable	–
*triloba*-complex*	67	*H. triloba, H. hageni, H. macilipennis, H. malenanda, H. efiel, H. yatrawalla*	yes	*gigantea* type	2/2	5/3	no/no	no/no	no	0
*H. yongma*	1	*H. yongma*	no	*unknown*	1/1	1/1	–	–	not applicable	–
*H. 677*	41	H. *677*	yes	*tibetana* type	1/1	1/1	yes/yes	yes/yes	yes	5
*H. 683*	8	*H. 683*	no	*tibetana* type	*martynovi*–clade	1/1	–	–	yes	–
*H. 685*	34	*H. 685*	yes	*tibetana* type	*martynovi*–clade	1/1	no/no	yes/yes	yes	3

### Morphology of *Himalopsyche* larvae

**Figures 3–6.**
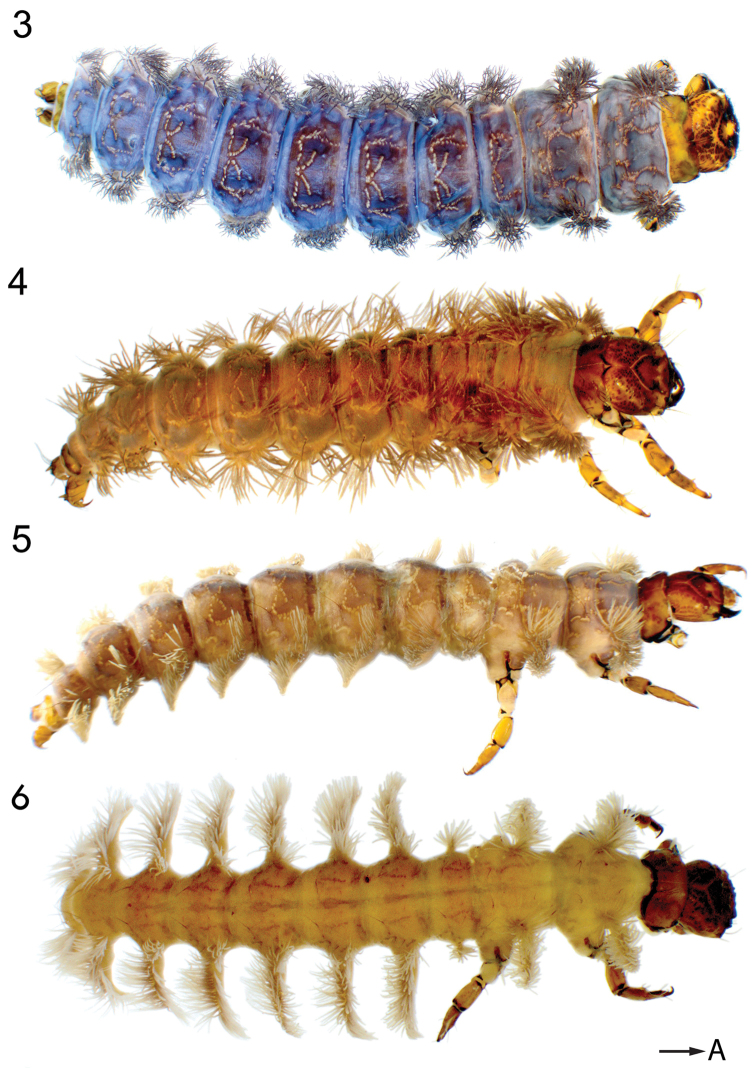
Habitus of *Himalopsyche* larvae. **3** *H. phryganea* **4** *H. gregoryi* **5** *H. japonica* **6** *H. sylvicola*. Arrow A points to anterior.

Synapomorphic larval characters of *Himalopsyche* according to Lepneva (1970), Ross (1956), Flint (1961), Ulmer (1957), and the present study are:

• Mandibles with prominent lateral protuberances (Figure 14, arrow)

• 2^nd^ and 3^rd^ leg with anterodorsal single coxal gills (e.g., Figure 18, arrow a, Figure 20, arrow a)

• Abdomen with prominent and complex gills consisting of a multitude of gill filaments positioned on several bases or a single base which can be slightly to distinctly protuberant (e.g. Figure 3).

• Anal proleg with two proximal accessory hooks fused with lateral sclerites (e.g. Figure 38, arrow d, Figure 41, arrow d).

Based on these characters, larvae of *Himalopsyche* can easily be differentiated from the closely related genera *Philocrena* and *Rhyacophila*. The larvae of the monotypic genera *Fansipangana* and *Phoupanpsyche* are unknown. Within *Himalopsyche*, the four different larval types can easily be differentiated based on distinct character states.

### *phryganea* type

**Figures 7–10.**
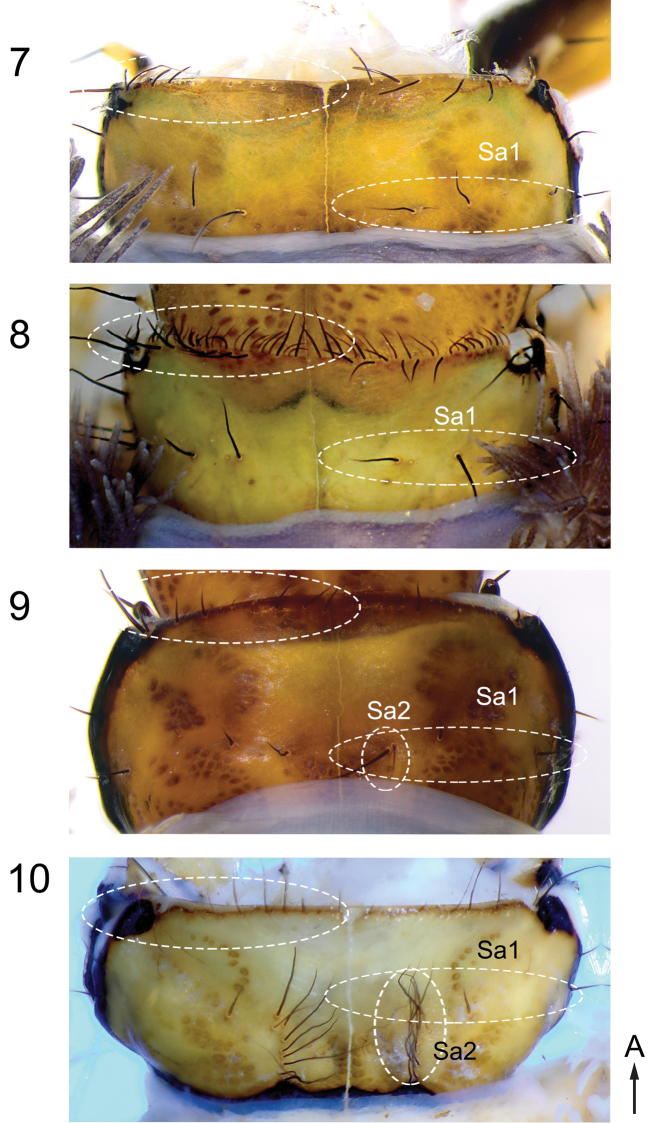
Pronotum of *Himalopsyche* larvae. **7** *H. phryganea* **8** *H. anomala* **9** *H. japonica* **10** *H. sylvicola*. Arrow A points to anterior.

**Figures 11–14.**
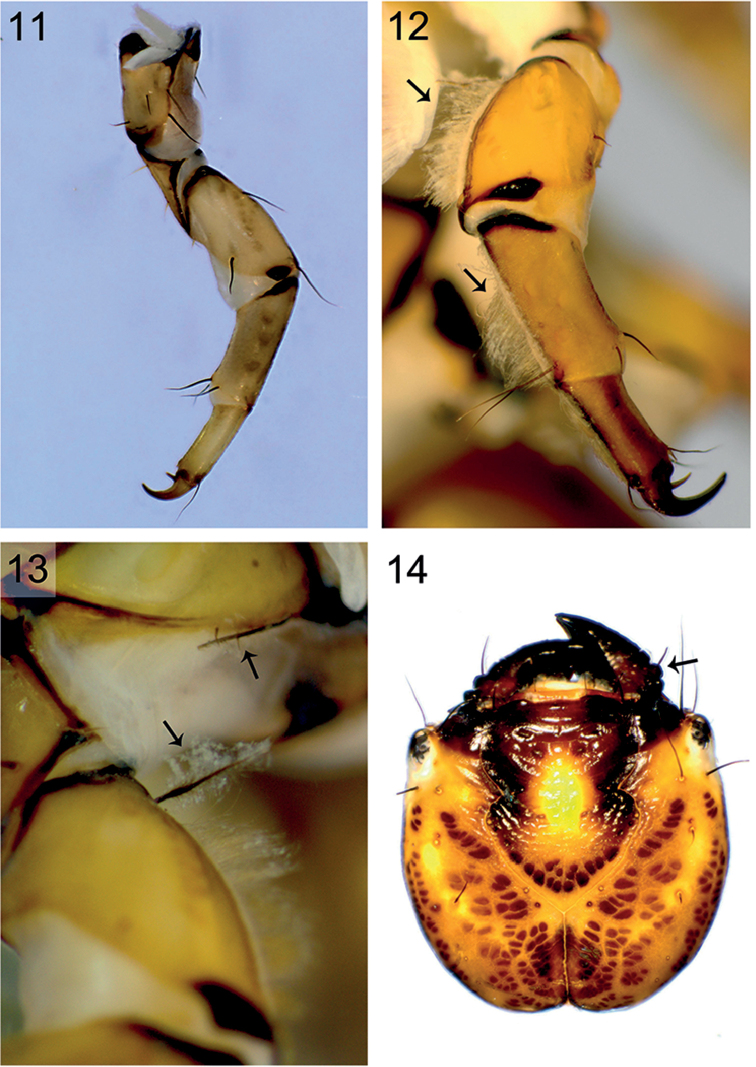
Mesolegs and head of *Himalopsyche* larvae. **11** Mesoleg of *H. gregoryi* **12** Mesoleg of *H. sylvicola*, arrows indicate dorsal fringe of setae on legs **13** Mesoleg of *H. sylvicola*. Arrows indicate pennate setae on coxa and femora **14** Head of *H. phryganea*, arrow points to lateral protuberances on mandible.

**Figures 15–22.**
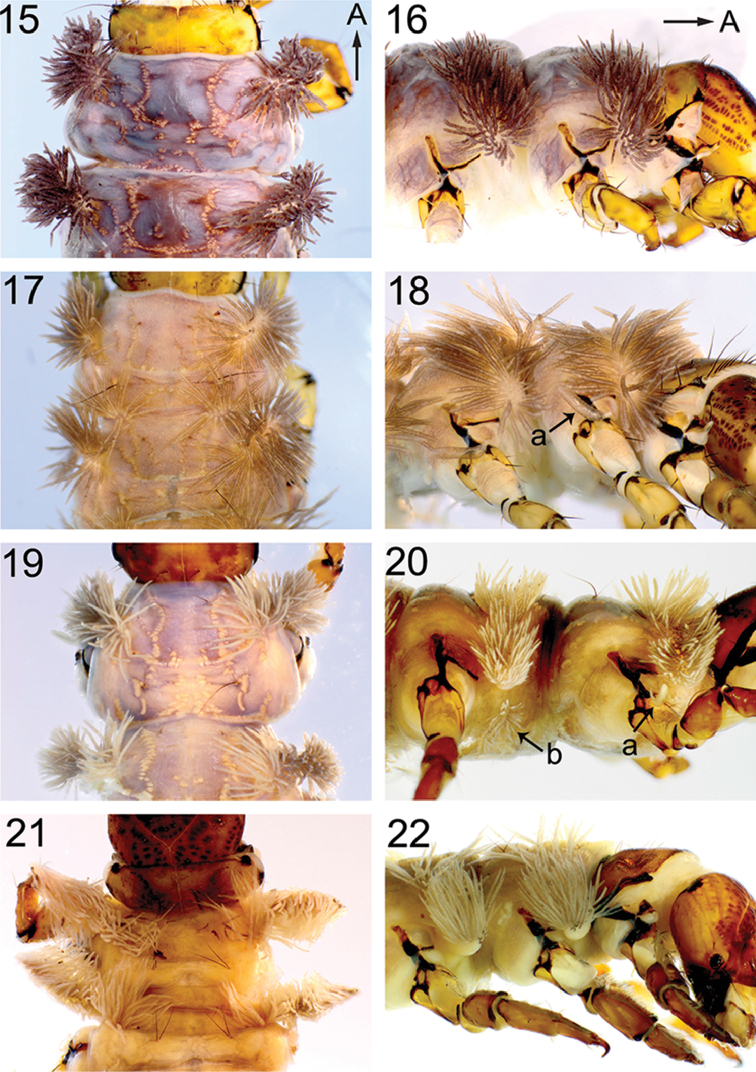
Thorax of *Himalopsyche* larvae in dorsal and lateral view. **15**, **16** *H. phryganea* **17, 18** *H. gregoryi*, arrow a points to anterodorsal single coxal gill **19**, **20** *H. japonica*, arrow a indicates anterodorsal single coxal gill; arrow b indicates the ventral gills **21**, **22** *H. sylvicola*. Arrows A points to anterior.

**Figures 23–28.**
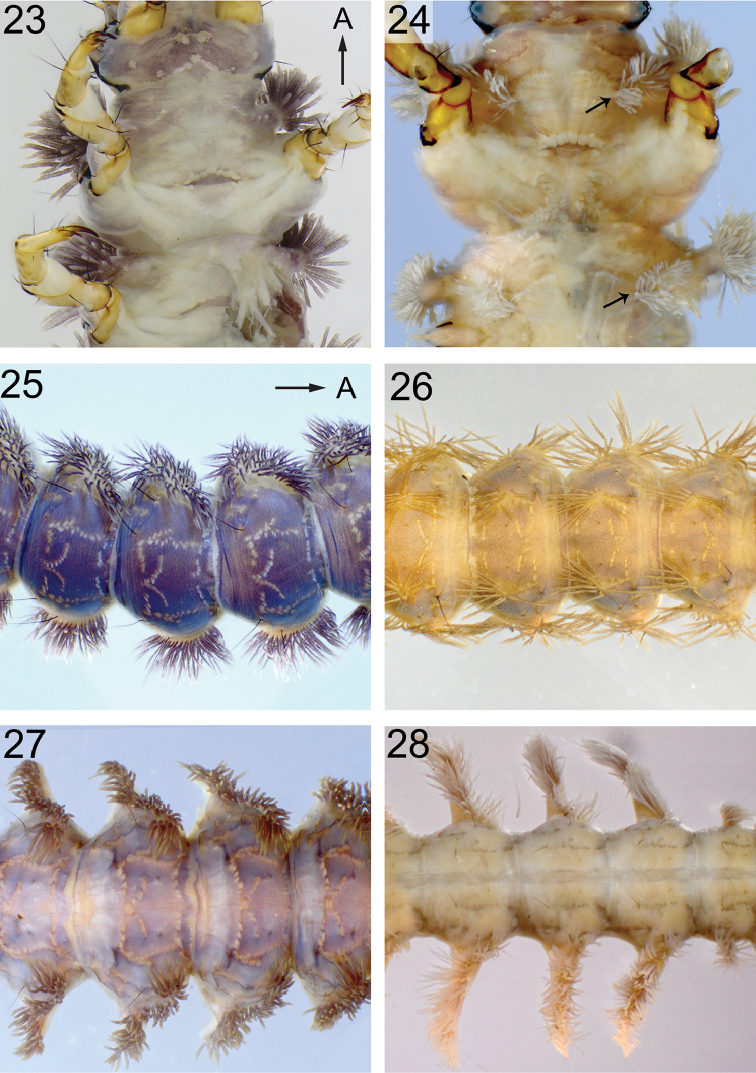
Thorax ventral, abdomen dorsal. **23** *H. gregoryi* **24** *H. japonica*, arrows indicate ventral gills **25** *H. phryganea* **26** *H. gregoryi*, **27** *H. acharai* **28** *H. sylvicola*. Arrows A points to anterior.

**Figures 29–36.**
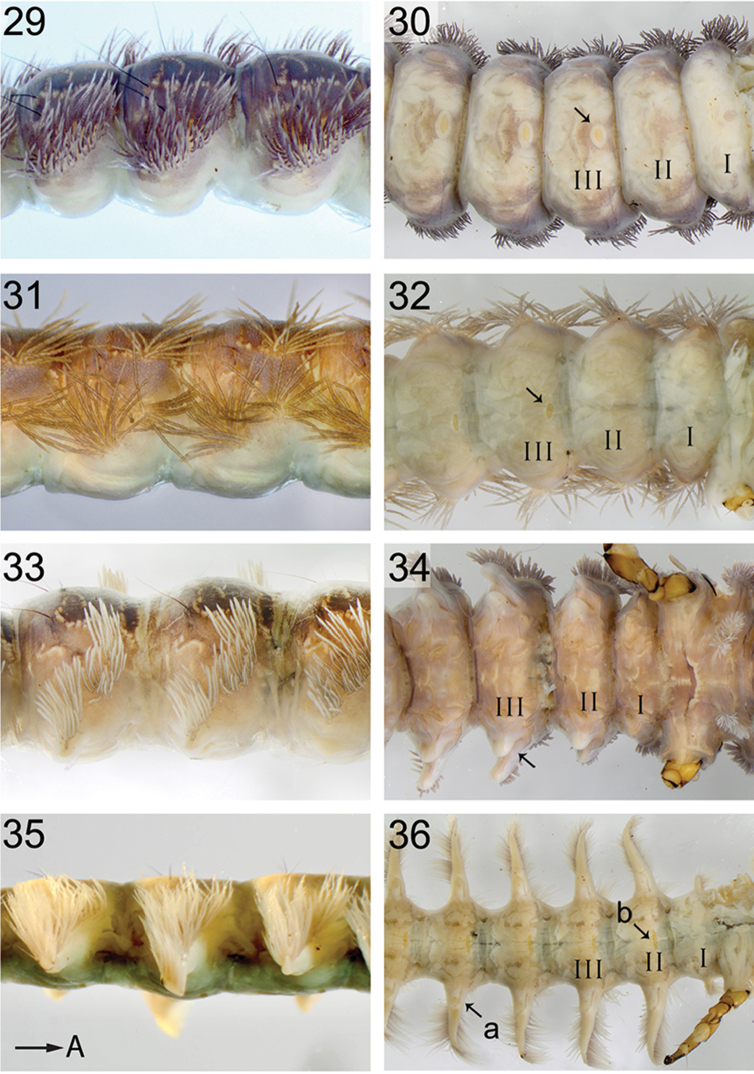
Abdomen lateral, ventral. **29**, **30** *H. phryganea*, arrow points to ventral medial sclerite **31, 32** *H. gregoryi*, arrow points to ventral medial sclerite **33**, **34** *H. japonica*, arrow points to ventral protuberance **35**, **36** *H. sylvicola*, arrow a indicates small rounded protuberance on lateral process, while arrow b indicates the ventral medial sclerite. Roman numbers indicate abdominal segments. Arrow A points to anterior.

**Figures 37–44.**
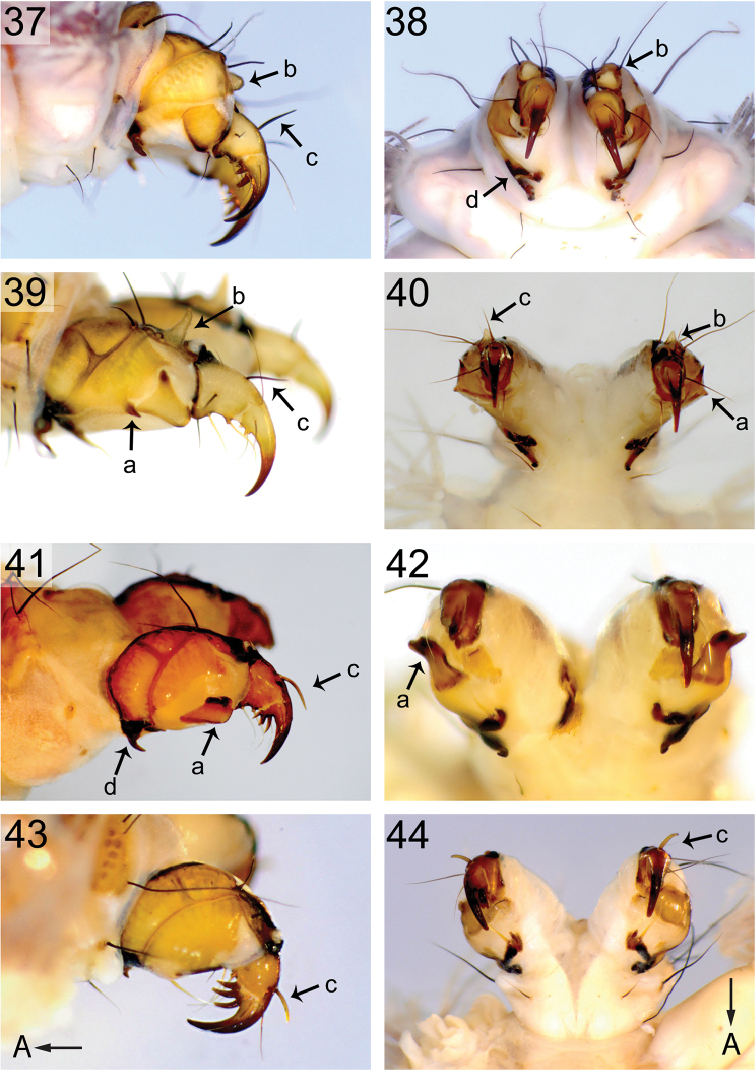
Anal prolegs, lateral, caudal. **37**, **38** *H. phryganea* **39** *H. gregoryi* **40** *H. tibetana* **41**, **42** *H. japonica* **43**, **44** *H. sylvicola*. Key: arrows **a** distolateral accessory hook. arrows **b** protuberance/hook on dorsal plate. arrows **c** dorsal spine on basal anal claw. arrows **d** proximal accessory hooks fused with lateral sclerites. Arrow A points to anterior.

*Himalopsyche phryganea* is the only species known with this larval type. Larvae of *H. phryganea* were described and illustrated by Flint (1961) and Wiggins (1996). Larvae of the *phryganea* type are characterised by the following set of characters:

Thorax. Pronotum with a single row of long dark setae along the entire anterior margin; short light recumbent setae concentrated at anterolateral pronotal edges; Sa1 present as a transversal band of 4–5 setae, Sa2 absent (Figure 7); legs without dorsal fringe of setae (Figure 11). Gills. Ventral gills at meso- and metathorax absent (Figure 23); thoracic and abdominal gills arranged on a single suboval, slightly protuberant base extending obliquely from anterodorsal to mediolateral position (Figs 3, 15, 16, 25, 29, 30). Abdomen. Abdomen without ventral protuberances (Figure 30); single ventral medial sclerite on abdomen III-VII, oval, transversally elongated (Figure 30, arrow). Anal prolegs. Stout, distolateral accessory hook absent, dorsal plate with rounded central protuberance (Figs 37–38, arrow b); dorsal spine on basal anal claw dark (Figure 37, arrow c).

### *tibetana* type

One species with larvae of the *tibetana* type has been described in the larval stage: *H. tibetana* (Graf and Sharma 1998, Type B). The following OTUs could be assigned to the *tibetana* type: *H. anomala*, *H. digitata*, *excisa*-complex, *H. gregoryi*, *martynovi*-complex, *platon*-complex, *H.*. sp. 1196 (L), and *H.*. sp. 1254 (L), *H. tibetana*, *H. 677*, and *H. 685*. Larvae of the *tibetana* type are characterised by the following set of characters:

Thorax. Pronotum with two rows of setae along the anterior edge, anteriormost row of setae short, light, recumbent and posterior row setae longer, black; Sa 1 present as a transversal band of 4–5 setae, Sa2 absent (Figure 8); legs without dorsal fringe of setae (Figure 11). Gills. Ventral gills at meso- and metathorax absent (Figure 23); thoracic gills arranged on anterodorsal and anterolateral bases, abdominal gills arranged on anterodorsal, anterolateral and posterolateral bases (Figs 4, 17, 18, 26, 31, 32); anterodorsal and anterolateral gill bases on abdomen I close, seemingly fused. Abdomen. Without ventral protuberances; single ventral medial sclerite on abdomen III-VII oval, transversally elongated (Figure 32, arrow). Anal prolegs. More elongated, with distolateral accessory hook (Figs 39–40, arrow a), dorsal plate with central hook (Figs 39–40, arrow b), dorsal spine on basal anal claw dark (Figs 39–40, arrow c).

### *japonica* type

Two species of the *japonica* type have been described in the larval stage: *H. acharai* by Thamsenanupap et al. (2005), and *H. japonica* by Saito (1965) and Tanida (1985). Larvae of the *japonica* type are characterised by the following set of characters:

Thorax. Pronotum with a single row of setae along the anterior edge; Sa1 present as a transversal row of 2–4 dark setae medially, Sa2 present as a group (sometimes arranged as sagittal band) of 2–4 setae (Figure 9); legs without dorsal fringe of setae (Figure 11). Gills. Ventral gills at meso- and metathorax present (Figure 24, arrows, Figure 20, arrow b); thoracic and abdominal gills arranged on two joint bases, one anterodorsal and one on a small lateral protuberance, extending posterolaterally (Figs 5, 19, 20, 27, 33, 34); abdomen I with gills on a single base only. Abdomen. With ventral protuberances (Fig. 34, arrow); ventral medial sclerites absent (Figure 34). Anal prolegs. Stout, distolateral accessory hook present (Figs 41–42, arrow a), dorsal plate flat without rounded central protuberance; dorsal spine on basal anal claw yellowish (Figure 41, arrow c).

### *gigantea* type

Larvae of this type have been described by Lepneva (1945, 1970), Schmid and Botosaneanu (1966), and Graf and Sharma (1998, Type A). Both Graf and Sharma (1998) and Schmid and Botosaneanu (1966) described larvae of unknown species identity. We assigned the following OTUs to the *gigantea* type: *H. sylvicola*, *triloba*-complex, and *H.*. sp. 1338 (L). Larvae of the *gigantea* type are characterised by the following set of characters:

Thorax. Pronotum with a single row of setae along the anterior edge; Sa1 present as a transversal band of 2–4 setae, Sa2 present as a sagittal band of 7–9 dark setae, prominent (10); legs with dorsal fringe of setae (Figure 12), with pennate setae on coxa and femora (Figure 13). Gills. Ventral gills at meso- and metathorax absent (Figure 23); thoracic and abdominal gills arranged on large lateral processes (conical processes *sensu* Graf and Sharma 1998), lateral processes slightly smaller on meso-and metathorax and distinctly smaller on abdomen I (Figs 6, 21, 22, 28, 35, 36); lateral processes with small rounded protuberances proximoventrally (Figure 36, arrow a). Abdomen. Without ventral protuberances; single ventral medial sclerite on abdomen II-VII suboval, thin, transversally elongated (Figure 36, arrow b). Anal prolegs. Stout, without distolateral accessory hook, dorsal plate flat without protuberance or hook (Figs 43–44); dorsal spine on basal anal claw yellowish (Figs 43–44, arrow c).

## Discussion

We used several life stage association strategies based on two genes in a comparative setting: PTP, GMYC and reciprocal monophyly. All strategies acknowledge a successful association in cases of sequence identity, but differ in that PTP and GMYC use branch lengths to estimate which clades represent distinct units, and that reciprocal monophyly requires congruence of gene trees. The importance of using more than one gene (Dupuis et al. 2012) was apparent in this study, as the two genes yielded somewhat different, albeit overall compatible, results. Delimitation results based on COI tended to split species into several units, especially with PTP, where CAD did not. This could be explained by the quicker coalescence time in COI, with its matrilineal inheritance leading to an effective population size 1/4 of that of nuclear genes. The PTP and GMYC methods search for the transition between inter- and intraspecific branching patterns, and if coalescence of COI lineages occurs within isolated populations, then the PTP and GMYC methods cannot distinguish between population and species signals. CAD was generally congruent with established taxonomy except in the species complexes, which were unresolvable by either gene. The major assumption, and limitation, of all association methods employed here is that they assume monophyletic gene trees. Closely related species may be difficult to separate genetically due to incomplete lineage sorting, and recent or ongoing gene flow (e.g., Kutschera et al. 2014). Therefore, recent divergences will always be problematic for PTP and GMYC analyses. GMYC has, for example, been shown to be an accurate and conservative method under conditions where effective sample sizes are low, and divergence times between species are high, since such conditions yield monophyletic species (Esselstyn et al. 2012, Fujisawa and Barraclough 2013). To resolve the species complexes more genes should be used, ideally under a multi-species coalescence approach, such as BPP (Yang 2015), or STACEY (Jones 2017). These methods combine data from several genes to estimate the species borders, accounting for incomplete lineage sorting and conflicting information among genes.

We observed clear morphological differences in morphology between the larval types. Within larval types, however, the examined material did not show any stable and reliable morphological characters to delimitate larvae at species-level. More material, particularly of last instar larvae, and ideally from numerous sites is required to better assess interspecific, intraspecific and ontological variation in the here described as well as other morphological characters. Only then will it be possible to assess if the observed morphological variation is useful for delimiting species in the larval stage.

## Conclusion

In this study, we present the characteristic morphological differences of four larval types of *Himalopsyche*. Life-stage association based on molecular data enabled us to do this, as it provided an OTU assignation for over 300 larvae. We found little or no morphological differences among species within the same type. Once we are able to better discern organisms at lower taxonomic rank (by morphology or molecular association, e.g., in high-throughput barcoding studies), aquatic insects and other benthic invertebrates can become much more valuable for biological monitoring in poorly studied regions. More generally, it is essential to be able to distinguish taxa at the lowest taxonomic resolution (i.e., to species) to understand their ecology and evolution. This is also of great relevance when developing tools to assess ecological status to ensure sustainable use and management of natural resources.

## Author contributions

AEH, SCJ, SP, and WG developed and planned the paper. AEH, SCJ, and SP conducted field work. AEH generated molecular, geographic and elevational data and performed molecular association analyses. AEH, SV and WG studied morphology of larvae; AEH drafted larval type descriptions with support from SV and WG. AEH and WG photographed larvae. AEH wrote the initial manuscript and all authors contributed substantially to further versions of the manuscript.

## Supplementary materials

Supplementary material 1 List of specimens Data type: speciemens data Explanation note: Table listing all specimens included in the study including their BOLD-IDs, species/OTU assignation, collection data, and haplotype numbers. This is an open access article distributed under the terms of the Creative Commons Attribution License (CC BY 4.0), which permits unrestricted use, distribution, and reproduction in any medium, provided the original author and source are credited.Anna E. Hjalmarsson, Wolfram Graf, Sonja C. Jähnig, Simon Vitecek, Steffen U. Pauls

Supplementary material 2 Figure S1. Life stage association results based on CAD Data type: molecular data Explanation note: The tree was generated in MrBayes (run1). Node values represent posterior probabilities. This is an open access article distributed under the terms of the Creative Commons Attribution License (CC BY 4.0), which permits unrestricted use, distribution, and reproduction in any medium, provided the original author and source are credited.Anna E. Hjalmarsson, Wolfram Graf, Sonja C. Jähnig, Simon Vitecek, Steffen U. Pauls

Supplementary material 3 Figure S2. Life stage association results based on COI Data type: molecular data Explanation note: The tree was generated in MrBayes (run1). Node values represent posterior probabilities. This is an open access article distributed under the terms of the Creative Commons Attribution License (CC BY 4.0), which permits unrestricted use, distribution, and reproduction in any medium, provided the original author and source are credited.Anna E. Hjalmarsson, Wolfram Graf, Sonja C. Jähnig, Simon Vitecek, Steffen U. Pauls
